# *Penicillium hordei* acidification precipitates *Bacillus subtilis* lipopeptides to evade inhibition

**DOI:** 10.1093/ismeco/ycaf172

**Published:** 2025-09-26

**Authors:** Manca Vertot, Morten D Schostag, Aaron J C Andersen, Jens C Frisvad, Carlos N Lozano-Andrade, Scott A Jarmusch

**Affiliations:** Department of Biotechnology and Biomedicine, Technical University of Denmark, Søltofts Plads, 2100 Kongens Lyngby, Denmark; Department of Biotechnology and Biomedicine, Technical University of Denmark, Søltofts Plads, 2100 Kongens Lyngby, Denmark; Department of Biotechnology and Biomedicine, Technical University of Denmark, Søltofts Plads, 2100 Kongens Lyngby, Denmark; Department of Biotechnology and Biomedicine, Technical University of Denmark, Søltofts Plads, 2100 Kongens Lyngby, Denmark; Department of Biotechnology and Biomedicine, Technical University of Denmark, Søltofts Plads, 2100 Kongens Lyngby, Denmark; Department of Biotechnology and Biomedicine, Technical University of Denmark, Søltofts Plads, 2100 Kongens Lyngby, Denmark

**Keywords:** Penicillium, Bacillus, organic acids, lipopeptides, precipitation, acidification, metabolomics, mass spectrometry imaging

## Abstract

Interkingdom interactions are crucial for community and ecosystem function; however, the secondary metabolites mediating interactions between plant beneficial bacteria and fungi remain understudied. Beneficial *Penicillium* and *Bacillus* species can individually suppress soilborne phytopathogens and promote plant growth. Here, we showed that *Penicillium hordei* and *Bacillus subtilis* co-culture led to precipitation of *B. subtilis* lipopeptides, observed as white lines in agar. Metabolomic analysis revealed that the presence of *B. subtilis* enhanced the production of fungal terrestric acid and its biosynthetic intermediates, which in turn induced lipopeptide precipitation, preventing *P. hordei* inhibition through chemical inactivation and physical barrier formation. Besides lipopeptide precipitation, terrestric acid-mediated acidification progressively reduced production of antifungal plipastatins. The lack of lipopeptide production permitted *P. hordei* to invade and overgrow the *B. subtilis* colony. We demonstrated that the white line phenomenon was conserved among closely related fungi via secretion of terrestric, fulvic, or barceloneic acids. Furthermore, terrestric acid at specific concentrations acted as a universal metabolite that drives *B. subtilis* lipopeptide precipitation even in distantly related fungi. This study provides new insights into acidification as a fungal defensive strategy that may promote co-existence with beneficial bacteria exhibiting strong antagonistic potential, thereby contributing to the formation of a stable rhizosphere community.

## Graphical Abstract

**Graphical Abstract:**
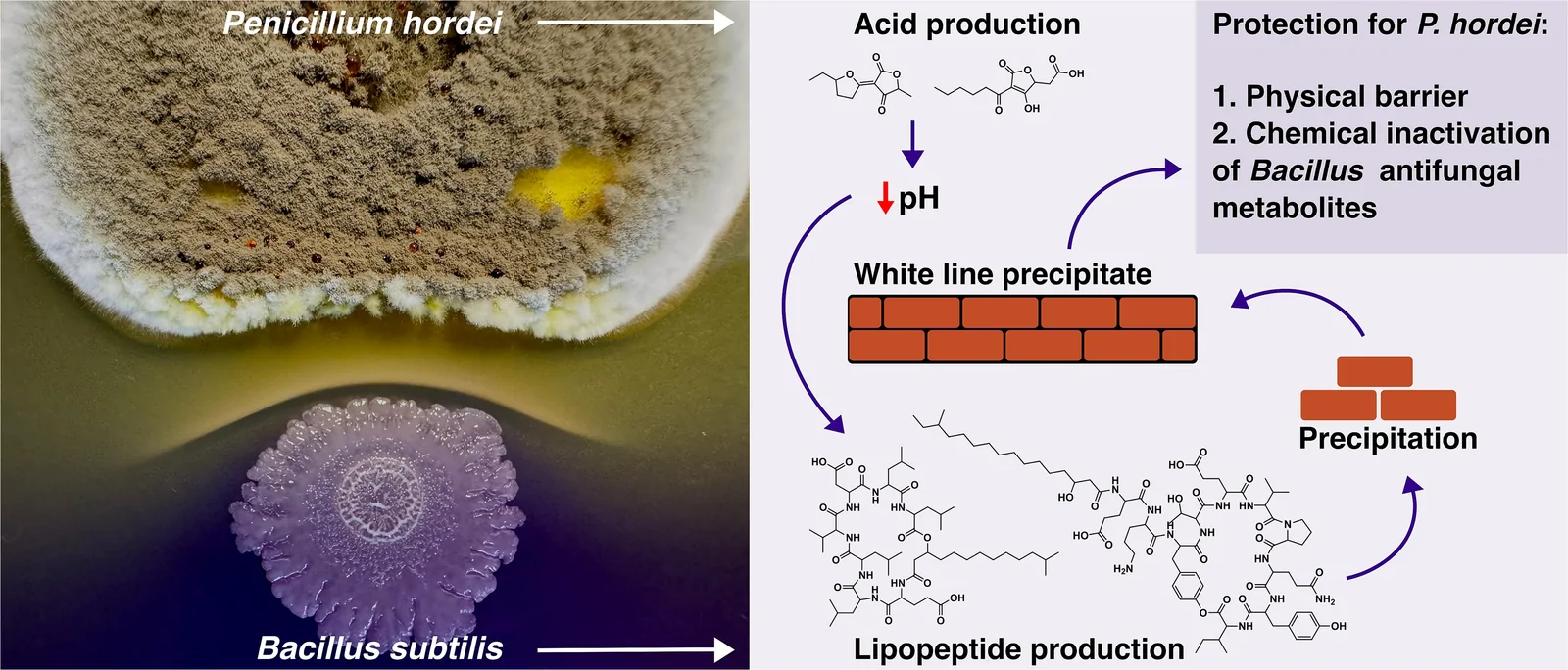

## Introduction

Fungi and bacteria are commonly found co-occurring in diverse habitats, including densely populated and nutrient-enriched layers of soil surrounding the plant roots (i.e. the rhizosphere) [1]. In the rhizosphere, they form complex microbial communities with other microorganisms and play a pivotal role in shaping community structure and function that ultimately affects plant health and ecosystem productivity [2, 3]. Their co-existence leads to extensive interkingdom interactions, ranging from cooperation to competition, which are mediated by small diffusible molecules known as secondary metabolites (SMs) [4–6]. While soil fungi and bacteria have been extensively studied individually for their production of SMs, little is known about the role these SMs play during interactions with one another.

*Penicillium hordei* resides in *Penicillium* section *Fasciculata*, series *Corymbifera* [7]*,* characterized by high extracellular enzyme activity, growth at low temperatures and close association with the rhizosphere of vegetables and flower bulbs. Unlike other members of this series, *P. hordei* does not cause blue mold storage rot, and it is uniquely associated with the rhizosphere of barley and other cereals [8, 9]. It is a prolific producer of pharmaceutically important ergot alkaloids, roquefortines and organic acids [10, 11]. Organic acids have been shown to confer a competitive advantage in producing microorganisms. For example, they can enhance the activity of cell wall degrading enzymes, stimulate the accumulation of SMs required for pathogenesis and counteract *Fusarium*-induced virulence [12–14]. However, their ecological relevance in microbial interactions is often overshadowed by more prominent SMs such as antibiotics.

*Bacillus subtilis* is a soil-dwelling bacterial species known to promote plant growth and act as a biocontrol agent against a wide range of organisms, such as bacteria [15], nematodes [16], and fungi [17, 18]. This capacity is attributed to its prolific biosynthetic potential to produce chemically diverse SMs, including cyclic lipopeptides (LPs), polyketides, and ribosomally synthesized and post-transcriptionally modified peptides. Among these, surfactins and plipastatins (LPs) have attracted the most attention due to their applications in biotechnological industry [19, 20]. From an ecological perspective, these SMs improve *Bacillus* fitness in the rhizosphere by enabling the bacterium to effectively colonize roots via biofilms which enables persistence in the niche [21]. Furthermore, they also serve as defense metabolites against cohabiting competitors, mediate microbial communication, and facilitate nutrient acquisition [22–24]. For example, surfactin-dependent motility allows *B. subtilis* to evade harmful metabolites produced by competitors [25, 26]. Similarly, Kiesewalter et al. showed plipastatins and surfactins are required to hinder the growth of *Botrytis cinerea* [27]*.* Microbial interactions are considered a major factor in modulating *Bacillus* lipopeptide production [28]. While research has primarily focused on interactions with phytopathogens, the role of SMs in interactions between *Bacillus* and epiphytic, non-pathogenic fungi remains largely unexplored.

In this study, we investigate the chemical interplay between prominent rhizosphere inhabitants, *P. hordei* and *B. subtilis*. By combining mass spectrometry-based metabolomics, mass spectrometry imaging (MSI) and molecular approaches, we demonstrated that *P. hordei* co-cultivation with *B. subtilis* leads to enhanced production of terrestric acid and its biosynthetic derivatives. Consequently, these acids drive *Bacillus* LP precipitation (observed as a white line) to confer a protective mechanism for the fungus. In addition to LP precipitation, terrestric acid-mediated acidification reduces the production of antifungal plipastatins in *B. subtilis*. The loss of LP production allows *P. hordei* to overgrow *B. subtilis,* highlighting the vital role of *Bacillus* lipopeptidome in sustaining competitive fitness to thrive upon co-culture with the fungus. We further reveal that the white line precipitation phenomenon is conserved among series *Corymbifera* through the production of different organic acids, as well as in distantly related fungi capable of producing terrestric acid, pointing to a widespread distribution of the defense strategy. Overall, our data suggests the complex interplay between these two beneficial soil microbes is mediated via SMs and may play an important role in the rhizosphere.

## Materials and methods

### Fungal and bacterial strains and culture conditions

Bacterial and fungal strains used in this study are listed in Table S1 and Table S2 in the supplemental material. *B. subtilis* strains were routinely grown in lysogeny broth (LB)-Lennox medium (10 g L^−1^ tryptone, 5 g L^−1^ yeast extract, and 5 g L^−1^ NaCl) at 37°C with shaking (180 rpm) and antibiotics when necessary. The final antibiotic concentrations for *B. subtilis* were: macrolide–lincosamide–streptogramin B (MLS) antibiotics (1 μg ml^−1^ erythromycin and 12.5 μg ml^−1^ lincomycin), spectinomycin (100 μg ml^−1^), and tetracycline (10 μg ml^−1^).

All fungal strains were routinely cultured on BD Difco potato dextrose agar (PDA) medium (4 g L^−1^ potato starch (infusion), 20 g L^−1^ dextrose, and 15 g L^−1^ agar). After incubation for 7 days at 25°C, spores were collected in 20% glycerol/water stock for subsequent experiments. To reduce terrestric acid production, *P. hordei* was grown on LB supplemented with maltose (20 g L^−1^) and agar (15 g L^−1^).

### Construction of *Bacillus subtilis co* P5_B1 *srfAC* ∆*ppsC*

Mutant strain was obtained using natural competence by transforming genomic DNA (gDNA) and selecting for antibiotic resistance as previously described [29]. Briefly, gDNA from a donor strain 3610 ∆*ppsC* was extracted using Monarch® Genomic DNA Purification Kit (NEB) following manufacturer’s instructions and 100 ng of gDNA was added to P5_B1 *srfAC* grown in 200 μl at 37°C in competence medium (80 mM K_2_HPO_4_, 38.2 mM KH_2_PO_4_, 20 g L^−1^ glucose, 3 mM tri-Na citrate, 45 μm ferric ammonium citrate, 1 g L^−1^ casein hydrolysate, 2 g L^−1^ K-glutamate, 0.335 μm MgSO_4_·7H_2_O, 0.005% [w/v] tryptophan). Cells were incubated with gDNA at 37°C for 4 h before 50 μl of the transformation mix was spread onto LB agar supplemented with spectinomycin and tetracycline and incubated at 37°C for 24 h. Mutant strain was validated by LC–MS analysis, confirming the absence of the following ions: plipastatin B2 (*m/z* 753.4286 [M + H]^2+^) and surfactin C14 (*m/z* 1022.6752 [M + H]^+^) (Fig. S1).

### Pairwise interactions on agar

For co-cultivation, overnight cultures of *B. subtilis* strains were normalized to an optical density at 600 nm (OD_600_) of 1 in LB. Subsequently, 1 μl of bacterial suspension and 1 μl of *P. hordei* suspension were applied at a 2.5 cm distance onto PDA and PDA supplemented with bromocresol purple (0.02 g L^−1^) and adjusted to pH 7. For each co-cultivation condition, a corresponding monoculture with the same inoculum was prepared. Three biological replicates of each condition were plated and incubated for 3 days, 5 days, 7 days, 9 days, and 12 days at 25°C in darkness.

The same co-cultivation process was applied for interactions with other LP-producing bacteria (Table S1) and *Penicillium* strains (Table S2). Plates were incubated for 9 days at 25°C in darkness.

### Extraction of secondary metabolites

To study SMs produced by *B. subtilis* strain*s* and *P. hordei* and how they vary in response to co-cultivation, we performed an untargeted time-course metabolomic analysis with four time points: days 3, 5, 7, and 9. For each time point, a 4 × 2 cm area of agar containing the co-cultures, and a 2 cm × 2 cm area for the monocultures were cut out, sliced into small pieces, and transferred into 15 ml tubes. The cultures were extracted with 1:3 isopropanol: ethyl acetate acidified with 1% formic acid and dried under nitrogen flow. For liquid chromatography–mass spectrometry (LC–MS/MS) analysis, the dried extracts were dissolved in 1.2 ml methanol (MeOH). The same extraction procedure was applied to interactions with other closely and distantly related *Penicillium* strains.

### Liquid chromatography–mass spectrometry data acquisition and processing

Crude extracts were analysed by an Agilent 1290 Infinity II UHPLC (Agilent Technologies; Santa Clara, CA) equipped with an Agilent Poroshell 120 Phenyl Hexyl column (1.7 μm, 150 × 2.1 mm) coupled to a Bruker TIMS TOF Flex. Chromatographic separation of extracts was achieved using the following solvent system: acetonitrile and water (both containing 20 mM formic acid), a flow rate of 0.35 ml min^−1^, a column temperature of 40°C and gradient over 14 min. The gradient started at 10% acetonitrile, increasing to 100% over 10 min, held at 100% for 2 min and returned to 10% acetonitrile over 0.1 min. The scan range was set to *m/z* 100–1400 and spectra were acquired at a rate of 10 Hz, top 3 precursor selection in a single MS1 which is followed by 9 MS/MS scans. The injection volume was 1 μl, and runs were conducted consecutively in negative and positive ionization mode. Automated DDA fragmentation was used with multiple collision energies (20 and 40 eV).

After LC–MS/MS acquisition, raw spectra were converted to .mzXML files using Bruker DataAnalysis and preprocessed using MZmine 3 [30]. Molecular networking and library search were performed within the GNPS platform [31] using feature-based molecular networking workflow (FBMN) [32].

### Sample preparation and matrix-assisted laser desorption ionization imaging mass spectrometry

Co-cultures and monocultures were prepared as described above onto a 10 ml PDA with bromocresol purple at 25°C in darkness. After 9 days of incubation, the aerial hyphae of *P. hordei* were gently removed with a cotton swab dampened in water (H_2_O). Afterwards, a region of agar containing either *P. hordei* and a *Bacillus* strain in co-culture, or a singly grown microbial culture, was excised and placed onto a Bruker IntelliSlide coated with a layer of glue applied with the ZIG 2-way glue pen (Kuretake Co., Ltd., Nara, Japan). Samples were dried for 2–5 h at 36°C followed by matrix application (30 mg ml^−1^ 2,5-dihydroxybenzoic acid, 50:50:0.1% H_2_O:MeOH:trifluoroacetic acid) for 12 passes using HTX-Sprayer (HTX Imaging, Chapel Hill, NC, USA) at 70°C. Samples were dried in a desiccator overnight prior to MSI measurement. The samples were then subjected to a Bruker TIMS TOF Flex (Bruker Daltonik GmbH, Bremen, GE) mass spectrometer for matrix-assisted laser desorption ionization imaging mass spectrometry (MALDI MSI) acquisition in positive MS scan mode with 60 μm raster width and a *m/z* range of 100–2000. Calibration was done using red phosphorus. For the acquisition timsControl software was used with the following settings:: Laser: imaging 60 μm, Power Boost 1.0%, frequency 10 000 Hz, scan range 56 μm in the XY interval, and laser power 80%; Tune: Funnel 1 RF 300 Vpp, Funnel 2 RF 300 Vpp, Multipole RF 300 Vpp, isCID 0 eV, Deflection Delta 70 V, MALDI plate offset 100 V, quadrupole ion energy 5 eV, quadrupole low mass 100 *m/z*, collision energy 10 eV, focus pre TOF transfer time 75 μs, pre-pulse storage 8 μs. After data acquisition, data was normalized to the total ion count and analysed using SCiLS software (version 2021b). Ion images were prepared and processed in R (V4.2.3) using a 50-ppm window for selected ions and FIJI (V2.16.0/1.54p) as previously described by Lyng et al. [33].

### Growth monitoring of *Bacillus* strains supplemented with terrestric acid

Growth experiments were performed in a 96-well microplate. Each well contained 160 μl of LB medium supplemented with 20 μl of terrestric acid at five concentrations (400, 200, 100, 50, and 25 μg ml^−1^), and 20 μl of either the *B. subtilis* wild type (WT) or its mutants were added. An untreated control containing only LB and bacterial suspension, as well as solvent control with MeOH and bacterial suspension, were included in the experiment. Four biological replicates were performed for each strain. The effect of terrestric acid was estimated by measuring the optical density at 600 nm every 5 min for 24 h with BioTek Synergy HTX Multimode Reader (Agilent Technologies, Santa Clara, CA), with continuous shaking at 150 rpm at 30°C.

### Statistical analysis

Data analysis and visualization were performed using R (V4.2.3) and the package ggplot2 [34]. Statistical analysis for pairwise comparison was explored via Student’s t or Welch’s test. For multiple comparisons (more than two treatments), one-way analysis of variance (ANOVA) followed by post hoc Tukey’s Honest Significant Distance (HSD) were performed. In all the cases, normality and equal variance were assessed using the Shapiro–Wilk and Levene tests. Statistical significance (α) was set at 0.05.

## Results

### Co-cultivation of *Penicillium hordei* and *Bacillus subtilis* leads to lipopeptide precipitation

**Figure 1:**
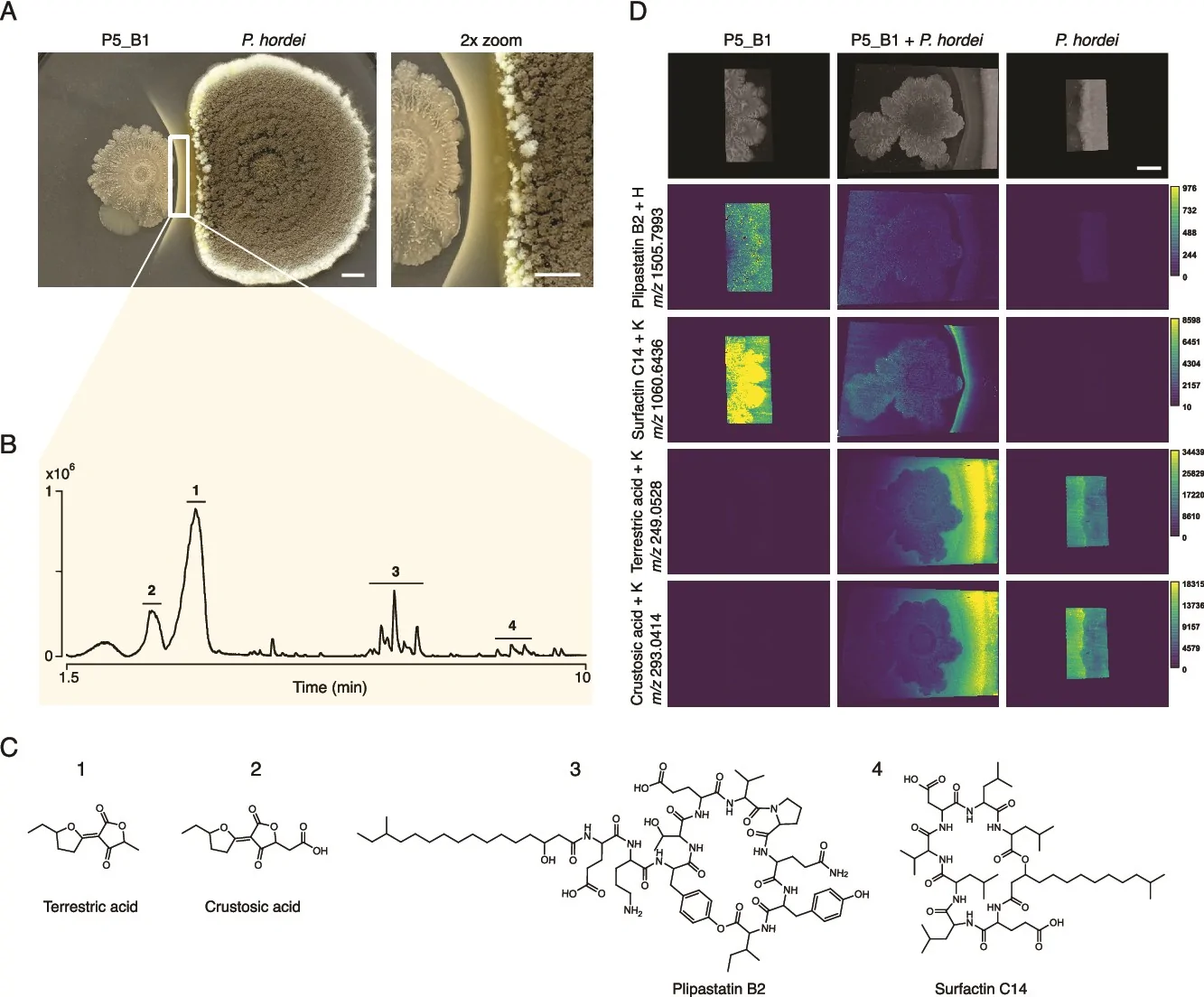
*P. hordei* induces precipitation of *B. subtilis* LPs. (A) Interaction between *P. hordei* (right colony) and *B. subtilis* P5_B1 (left colony) on PDA plate results in white precipitate formation. Close-up (2×) of interacting area is shown on the right, and image was taken on day 12. The square indicates the area selected for LC–MS/MS analysis shown in panel B. Scale bar = 5 mm. (B) Base peak chromatogram in the positive mode of the crude extract from the white precipitate region area showing the accumulation of fungal organic acids and bacterial cyclic LPs. The y-axis represents peak intensities. Numbers correspond to the following SMs: 1: terrestric acid; 2: CA; 3: plipastatins; 4: surfactins. (C) Chemical structures of SMs detected from the white line precipitate region. Surfactin C14 and plipastatin B2 are shown as isoform representatives for surfactins and plipastatins. (D) MALDI-MSI reveals the spatial distribution of selected ions in the interaction and corresponding monocultures on day 9. Ion images are displayed with a 50-ppm window using viridis color scheme. Colored scale bars besides ion images indicate a total ion count. The complete MALDI-MSI data set is shown in Fig. S3. Scale bar = 5 mm (top-right image).

To explore the chemical interplay driving cross-kingdom interactions between fungi and bacteria, we co-cultivated two beneficial wild rhizosphere isolates, *P. hordei* and *B. subtilis* P5_B1, on PDA. After 6 days of co-cultivation, a white precipitate formed near the *B. subtilis* colony. The precipitation progressively increased over time, expanding outward from the interface and gradually surrounding the *Bacillus* colony (Fig. 1A, Video S1). To characterize the chemical composition of the white precipitate, the agar between the colonies was excised, extracted and analyzed by LC–MS/MS. Initial profiling of crude extracts revealed a mixture of fungal organic acids and *Bacillus* cyclic LPs. Mass spectrometry-based fragmentation library matching and feature-based molecular networking identified the organic acids as terrestric acid (TA) (i) and its biosynthetic byproducts: dehydroterrestric acid, crustosic acid (CA) (ii) and viridicatic acid (VA) (Fig. 1B, Fig. S2). Similarly, the *B. subtilis* LPs were identified as plipastatin (plipastatin A2, B1, B2) (iii) and surfactin isoforms (surfactin C13-C15) (4) (Fig. 1B, Fig. 1C), all common LPs produced by several strains of *B. subtilis* [20].

To resolve the spatial distribution of the SMs observed in the bulk extraction, the interacting *P. hordei* and *B. subtillis* P5_B1 colonies, along with their corresponding monocultures, were excised from thin agar plates on day 9 and subjected to MALDI-MSI. MSI confirmed the accumulation of *B. subtilis* LPs (surfactins, plipastatins, and gageopeptides) in the region corresponding to the white line precipitate (Fig. 1D). Specifically, ions at *m/z* 1030.6360, 1060.6436, and 1074.6563 were identified as potassium adducts of surfactins with fatty acid chain lengths of C13, C14, and C15, respectively (Fig. 1D, Fig. S3). These surfactin isoforms exhibited the highest signal intensity at the white precipitate region and in the *B. subtilis* colony, particularly on the side facing away from the interaction zone. Similarly, isoforms belonging to the plipastatin family (*m/z* 1491.8231 and 1505.7993) and the linear LP gageopeptides (*m/z* 702.4742, 738.4721, and 754.4456) followed the same spatial distribution pattern. However, plipastatin relative accumulation in this area was less pronounced compared to the surfactins (Fig. 1D, Fig. S3).

TA and CA were also detected, showing enhanced production upon interaction with *B. subtilis*. These ions diffused from the *P. hordei* colony across the *B. subtilis* colony, extending nearly to its far edge (Fig. 1D). The highest signal intensity was observed at the interface, spanning from the fungal colony to the white line precipitate. The presence of LPs in this region, along with the high relative concentration of TA and its biosynthetic byproducts, led us to hypothesize that organic acids secreted by *P. hordei* induce the precipitation of antifungal LPs produced by *B. subtilis*, thereby conferring a protective effect on the fungus.

### Lipopeptide precipitation induced by *P. hordei* acidification prevents fungal inhibition

We visually assessed the ability of *P. hordei* to alter the pH of culture media using two pH indicators, bromocresol purple (purple when pH is above 6.8 and yellow below 5.2) and bromocresol green (blue to yellow below pH 3.8). When cultured alone on these plates, *P. hordei* triggered a rapid and progressive drop in pH on bromocresol purple within 7 days (Fig. S4A). By day 7, bromocresol green plates confirmed that *P. hordei* lowered the pH of the medium below 3.8, demonstrating a strong acidification capacity (Fig. S4B). This observation aligns with our previous LC–MS/MS analyses, which identified TA as the primary small organic acid secreted by *P. hordei* in interaction with *B. subtilis* (Fig. 1B).

**Figure 2:**
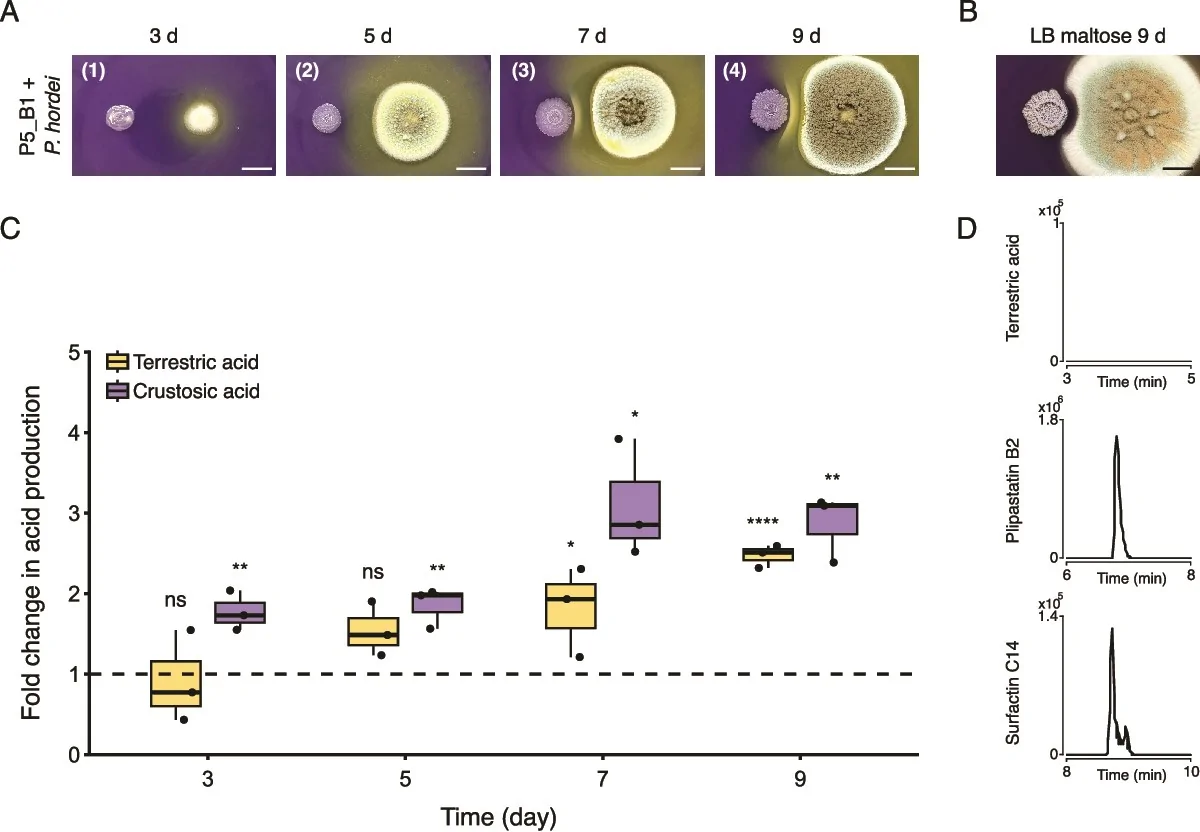
Enhanced production of organic acids by *P. hordei* drives *B. subtilis* lipopeptide precipitation to prevent fungal inhibition. (A) Time-course of the interaction between *P. hordei* and *B. subtilis* on pH indicator plates. Numbers in the upper left corner correspond to the different phases of interaction: (i) before white line formation; (ii) before white line formation but with reduced distance between colonies; (iii) onset of lipopeptide precipitation, and (iv) accumulation of LPs, leading to the formation of a thicker white precipitate. Scale bar = 1 cm. (B) *P. hordei* failed to induce lipopeptide precipitation when acid production was highly reduced in interaction on LB containing 20% maltose by day 9. Scale bar = 1 cm. (C) impact on terrestric (yellow) and crustosic (purple) acid production by *P. hordei* in response to *B. subtilis* over time (days 3, 5, 7, 9) on pH indicator plates. Fold changes were calculated based on relative quantification of the acids by LC–MS (peak area) in co-culture compared to monoculture of *P. hordei* (fold change = 1, dashed line). Box plots were generated from three biological replicates (*n* = 3), where whiskers span from the minimum to maximum values, and black line inside the box indicates median. Statistical significance for terrestric acid production was calculated using Student’s *t*-test, while Welch’s *t*-test was used for CA production, where “ns” denotes no significant difference; ^*^P < .05; ^**^, *P* < .01; ^***^P < .001; ^****^P < .0001. (D) Extracted ion chromatograms of terrestric acid (top), plipastatin B2 (middle) and surfactin C14 (bottom) detected in *P. hordei* and P5_B1 co-culture on LB-maltose.

Further, we explored how acid production drives the LP precipitation by combining the use of pH indicators plates with untargeted metabolomics analysis, allowing for visual inspection of LP precipitation and relative quantification of SMs in the interacting region over time (Fig. 2A). Here, the four main organic acids produced by *P. hordei* steadily increase over time, peaking on day 7 and 9 and reaching concentrations around 3-fold higher than in monocultures (TA on day 9, *t*-test, *P* = 5.32 × 10^−5^; CA on day 9, *t*-test, *P* = 2.17 × 10^−3^) (Fig. 2C, Fig. S4).

We corroborated such observation by propagating both interacting partners on LB agar plates supplemented with 20% maltose, a growth condition where acid production is highly reduced, but the LP production is maintained to a level similar to the ones obtained on PDA plates (Fig. 2B, Fig. 2D). Under this condition, we demonstrated that LP precipitation via acid production confers a protective mechanism for *P. hordei.* In the absence of such precipitation, *P. hordei* growth was impaired by *B. subtilis*, as evidenced by the clear inhibition halo (Fig. 2B).

### *Corymbifera* series Penicillia induce lipopeptide precipitation via production of different organic acids

**Figure 3:**
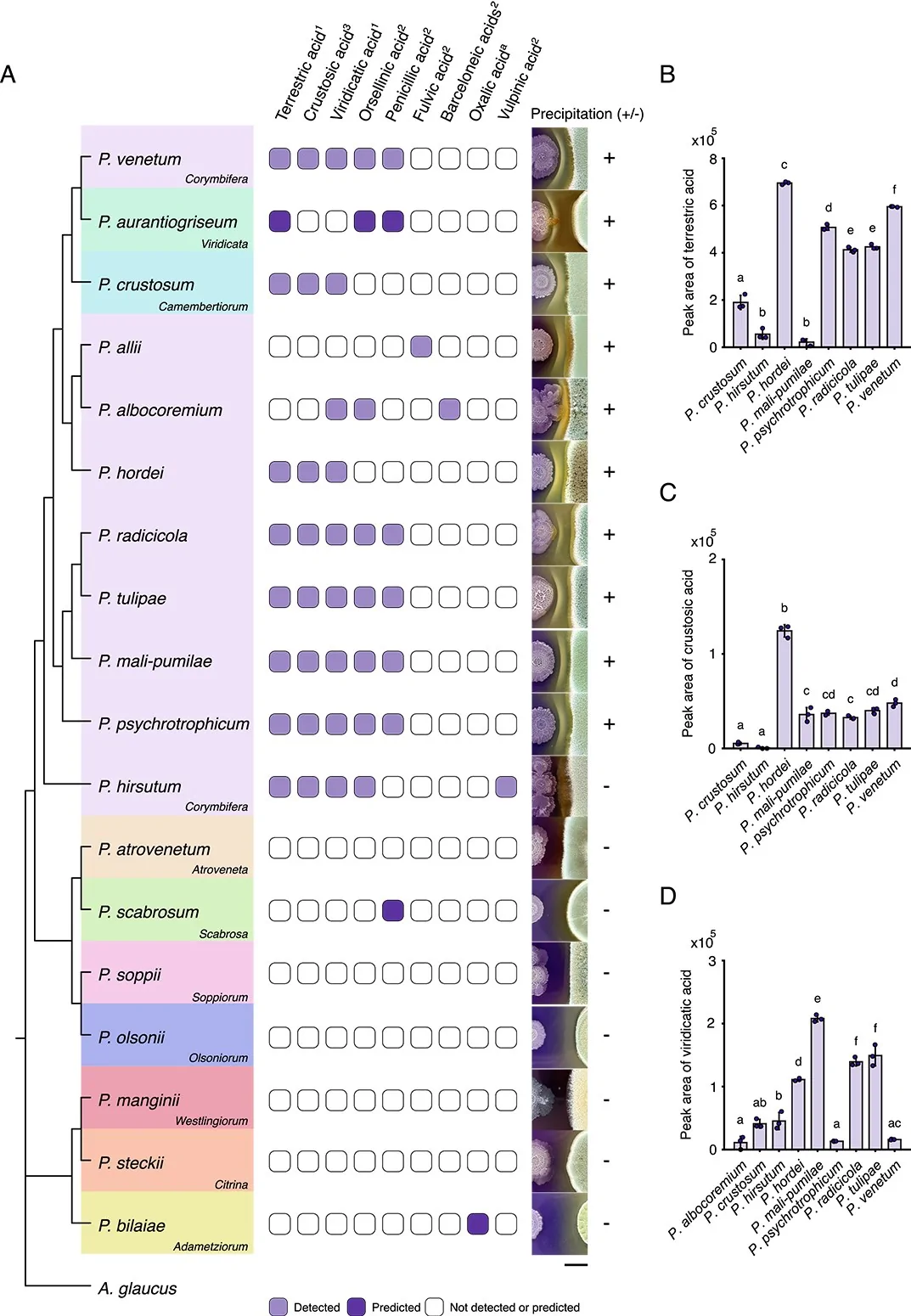
*Corymbifera* series Penicillia induce lipopeptide precipitation through secretion of distinct organic acids. (A) Closely related *Corymbifera* species except for *P. hirsutum* induced lipopeptide precipitation via production of terrestric, fulvic, or barceloneic acids. Among distantly related fungi, *P. crustosum and P. aurantiogriseum* were also able to form the white precipitate through the production of terrestric acid*.* The phylogenetic tree was built based on calmodulin sequences of interacting type fungal strains using maximum likelihood method and Kimura (1980) 2-model [64]. The phylogram was rooted with *Aspergillus glaucus* as an outgroup and the analysis was conducted in MEGA12 [65]. Fungal species are color-coded according to their taxonomic series, indicated in the lower-right corner of each colored box. Light purple squares indicate acids detected by LC–MS, dark purple squares indicate acids predicted to be produced by certain fungal species based on chemotaxonomic survey, and white squares indicate that no acids were detected or predicted. Numbers next to acids refer to identification level (1–3): (1) reference standard, (2) confident match based on MS/MS, and (3) MS only [66]. The letter a indicates compounds identified as a chemotaxonomic marker based on a chemotaxonomic survey. Images on the right display the presence (+) or absence (−) of the white line precipitation between *B. subtilis* P5_B1 and the respective fungal species on pH indicator plates. Scale bar = 5 mm. (B–D) Production of terrestric, crustosic, and VAs of *Corymbifera* and *Camembertiorum* species varied in co-culture with *B. subtilis*. Acid production was quantified by LC–MS (peak area) and mean values were calculated from three biological replicates (*n* = 3). Error bars represent standard deviation; grouping letters are from ANOVA with Tukey–Kramer’s post hoc test, where different letters within plots indicate a statistically significant grouping on day 9 (*P* < .05).

To investigate how widespread the white line precipitation phenomenon is, we co-inoculated *B. subtilis* with eight fungal strains closely related to *P. hordei* from the *Corymbifera* series on pH indicator plates, as described above. To capture a broad range of fungal diversity, we also included distantly related fungal species from *Penicillium* series *Adametziorum*, *Viridicata*, *Atroveneta*, *Scabros*a, *Soppiorum*, *Olsoniorum*, *Camembertiorum*, *Citrina*, and *Westlingiorum* [7]. All the *Corymbifera* species tested, except *Penicillium hirsutum*, induced *B. subtilis* LP precipitation, mirroring the abundance and spatio-temporal precipitation patterns observed in the interaction with *P. hordei* (Fig. 3A). A targeted metabolomics approach utilizing known chemotaxonomic markers [7, 35, 36], revealed that fungi capable of inducing the white precipitate produced various organic acids, including TA, CA, and VA (Fig. 3A). Within the *Corymbifera* series, *Penicillium albocoremium* induced white line precipitation by predominantly producing barceloneic acid A, whose concentration was approximately 17-fold higher than VA. Two other *Corymbifiera* species exhibited contrasting precipitation patterns: *P. hirsutum* did not induce LP precipitation, consistent with its low TA and CA production compared to *P. hordei* (one-way ANOVA, *P* = 2.3 × 10^−14^) (Fig. 3B–D), whereas *Penicillium allii* triggered LP precipitation despite lacking TA and its biosynthetic derivates and producing only fulvic acid (Fig. 3A and Fig. S6).

Among distantly related species tested, only *Penicillium crustosum* (*Camembertiorum* series) and *Penicillium aurantiogriseum* (*Viridicata* series), both members of the *Fasciculata* section, were able to induce LP precipitation through the production of TA. Other distantly related *Penicillium atrovenetum*, *Penicillium scabrosum*, *Penicillium olsonii*, *Penicillium soppii*, *Penicillium manginii*, *Penicillium steckii*, and *Penicillium bilaiae* were all able to modify the pH of the interaction plates yet failed to precipitate LPs. The chemotaxonomic survey showed that apart from *P. scabrosum* producing penicillic acid, none of these species are known to produce the organic acids detected in the *Corymbifera* species. Overall, these data confirm that fungal species capable of inducing white line precipitation also produce higher levels of multiple organic acids, linking acid production to the observed white line phenomenon.

### Plipastatin production is reduced by terrestric acid-mediated acidification

**Figure 4:**
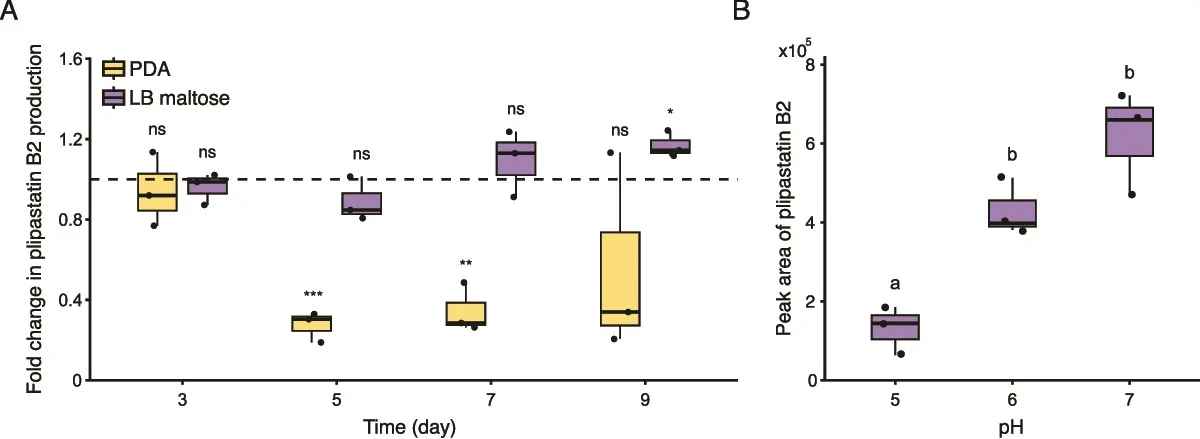
Plipastatin production is reduced by fungal-mediated acidification. A impact on plipastatin B2 production in the presence of fungal acidification on PDA (yellow) and under highly reduced acidification on LB-maltose (purple). Fold changes were calculated based on relative quantification of the plipastatin B2 by LC–MS (peak area) in co-culture compared to monoculture of *B. subtilis* P5_B1 (fold change = 1, dashed line). Box plots were generated from three biological replicates (*n* = 3), whiskers span from the minimum to maximum values, and black line inside the box indicates the median. Statistical significance was calculated using Student’s *t*-test where “ns” denotes no significant difference; ^*^P < .05; ^**^P < .01; ^***^P < .001. B Plipastatin B2 production by *B. subtilis* P5_B1 decreased when grown alone on PDA at low pH values (pH 5, 6 and 7) on day 7. Plipastatin B2 production was quantified by LC–MS (peak area) from three biological replicates (*n* = 3) per each pH value; grouping letters are from ANOVA with Tukey–Kramer’s post hoc test, where letters within a plot indicate a statistically significant grouping (*P* < .05).

Once we determined the direct role of *P. hordei* acidification in causing LP precipitation, we investigated its consequences for *B. subtilis,* given that LPs are crucial defensive SMs in bacilli. To do so, we confronted *P. hordei* with a panel of *B. subtilis* strains on pH indicator plates: the parental WT, *sfp* (impaired in non-ribosomal peptide synthesis), *srfAC* (surfactin-deficient), and the double mutant *srfAC* Δ*ppsC* (lacking both plipastatin and surfactin). In co-cultures with the WT strain, plipastatin production declined significantly, dropping to about 30% of monoculture levels by days 5 and 7 (*t*-test, *P* = 4.510^−4^ and *P* = 5.28 × 10^−3^) (Fig. 4A). When acidification was reduced on LB-maltose medium, plipastatin levels rose on day 9 (t-test, *P* = .03), and *B. subtilis* more effectively inhibited *P. hordei*, suggesting that fungal mediated acidification impacts plipastatin production (Fig. 4A).

To confirm that acidification leads to reduced plipastatin production, we cultivated the *B. subtilis* WT strain alone on plates with different starting pH values (pH 5, 6, and 7). On day 5, plipastatin levels were comparable across all pH conditions (one-way ANOVA resulted in nonsignificant differences between groups) (Fig. S7). In contrast, on day 7, plipastatin levels at pH 5 were significantly reduced than those at pH 6 and 7 (one-way ANOVA, *P* = 1.8 × 10^−4^), whereas no significant difference was observed between pH 6 and 7 (Fig. 4B).

We next asked whether the reduction in plipastatin production was a general response to low pH, regardless of which acid-producing fungal species *B. subtilis* was confronted with. Closely related fungi and *P. crustosum* were co-cultivated for 9 days in darkness as described above. Here, plipastatin production was strongly reduced during interactions with TA-producing species, including *P. venetum*, *P. tulipae*, *P. radicicola*, and *P. psychrotrophicum* (*t-*test, *P* = .035, *P* = .005, *P* = .013, *P* = .017) (Fig. S8). However, enhanced plipastatin production was observed upon co-cultivation with *P. allii* and *P. albocoremium*, both species that lack TA production but instead produce fulvic and barceloneic acids, respectively (Fig. 3A, Fig. S6). TA and its associated intermediates show the ability to affect plipastatin production, whereas other organic acids fail to do so despite their induced LP precipitation.

### Surfactin-mediated precipitate forms a shared protective physical barrier

**Figure 5:**
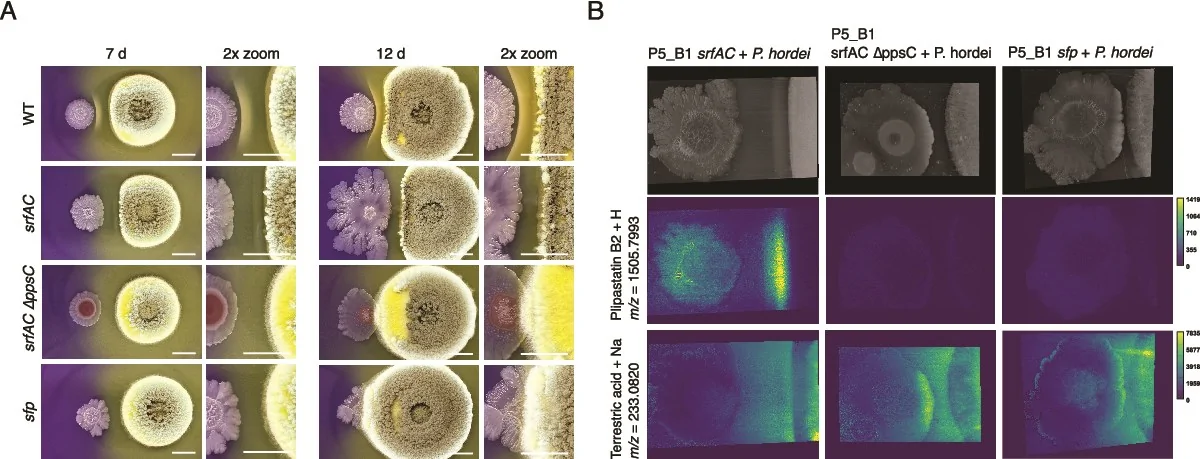
Lack of lipopeptide production by *B. subtilis* allows *P. hordei* to invade and overgrow *the Bacillus* colony. (A) Time-course of interactions between *P. hordei* and *B. subtilis* strains: WT *srfAC* (surfactin-deficient), *srfAC* Δ*ppsC* (double mutant lacking both plipastatin and surfactin), and *sfp* (impaired in non-ribosomal peptide synthesis). Interactions were observed on days 7 and 12 using pH indicator plates*.* A close-up (2×) of the interacting area is shown on the right of each image. Left colonies are *B. subtilis* strains and right colonies are *P. hordei*. Scale bar = 1 cm. (B) MALDI-MSI reveals the spatial distribution of plipastatin B2 and terrestric acid in the interactions with *B. subtilis* mutants on day 9. Ion images are displayed with a 50-ppm window using viridis color scheme. Colored scale bars besides ion images indicate a total ion count. Scale bar = 5 mm (top-right image).

We then examined the contribution of surfactin to the white precipitate formation and its respective role in the interaction. In contrast to plipastatin, fungal acidification had no significant impact on surfactin production in WT strain (Fig. S9). When *P. hordei* was confronted with *srfAC* strain, a white line precipitate formed near the fungal edge, but it was less intense compared to that formed in the interaction with the WT strain (Fig. 5A). As expected, MALDI-MSI analysis revealed accumulation of only plipastatin isoforms (*m/z* 1477.8288, 1491.8231, and 1505.7993 were identified as proton adducts of plipastatin B2, plipastatin A2, and plipastatin B1) at the white precipitate region. These ions showed the highest signal at the white precipitate region, with a reduced signal on the outer side of the *Bacillus* colony, facing away from the fungus (Fig. 5B, Fig. S10). The loss of surfactin production resulted in stronger inhibition of the fungus compared to the WT strain, as indicated by denser and elevated aerial hyphae and the accumulation of brown exudate droplets at the interface (Fig. 5A). Compared to the WT strain, *srfAC* strain was more affected and exhibited an increased swarming motility between day 7 and day 12. Altogether, these observations confirm the vital role of surfactins in a white precipitate formation, serving as a physical barrier that provides protection for both interacting partners.

### *Bacillus subtilis* relies on lipopeptide production for defense

Since the *srfAC* strain was still able to resist fungal takeover, we co-cultivated *P. hordei* with *srfAC* Δ*ppsC* and *sfp* strains. In both cases, *P. hordei* was able to partially invade the frontline of *Bacillus* colony after 12 days and after 15 days, nearly the entire colony was covered with *P. hordei* mycelium (Fig. 5A, Video S2). Compared to interactions with the WT and *srfAC* strains, the distribution pattern of TA (*m/z* 233.0820 [M + Na]^+^) was altered with increased signal intensity at the frontline of the *Bacillus* colony, suggesting localized accumulation (Fig. 5B). This was further supported by the yellow coloration of the outer edge of *Bacillus* colony at the interface. Despite lacking LP production, the mutant strains retained the ability to enhance TA production in the fungus. Both the *srfAC* Δ*ppsC* and *sfp* strains increased TA levels to a similar extent as the WT strain on day 9, thereby ruling out LPs as the inducing signals (Fig. S11). Interestingly, without the LPs, *B. subtilis* lacks the ability to buffer the medium, which allows for TA to diffuse through the bacterial colony. Subsequently, to evaluate if TA is toxic for *B. subtilis*, we tested five different concentrations of pure TA (400, 200, 100, 50, and 25 μg ml^−1^) on *B. subtilis* strains (WT, *srfAC,* and *sfp*) and their growth was monitored over 24 h period. The results showed that TA at all tested concentrations had no inhibitory effect on *B. subtilis* growth (Fig. S12).

Having established that white line precipitation is required for *B. subtilis* to prevent its overgrowth, we next examined whether *P. hordei* could cause the same effect when confronted with other LP-producing bacteria. While *P. hordei* was able to precipitate LPs in co-culture with *Bacillus velezensis* and *Bacillus tequilensis*, it failed to do so with all tested *Pseudomonas* strains (Fig. S13). In contrast to bacilli, *Pseudomonas* strains were able to acidify the culture medium and did not exhibit any deleterious effects on fungal growth.

## Discussion

**Figure 6:**
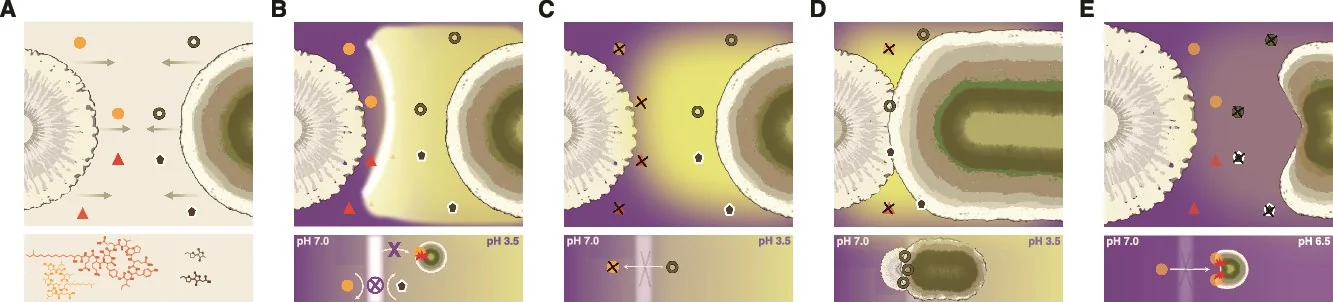
Graphical summary of outcomes and mechanisms in *P. hordei*–*B. subtilis* interactions. (A) Upon interaction, *B. subtilis* produces LPs, including plipastatin (red) and surfactin (orange), while *P. hordei* enhances production of organic acids, particularly terrestric acid (dark green) and CA (brown). (B) Fungal acidification drives precipitation of *B. subtilis* LPs, which leads to the formation of the white line on agar plates. The white line precipitate serves as a physical barrier, protecting both *P. hordei* and *B. subtilis* by restricting metabolite diffusion toward each other. In addition, by precipitating antifungal plipastatins, *P. hordei* prevents them from reaching the fungal colony and exerting their activity. (C) When the *B. subtilis sfp* mutant is unable to produce LPs, *P. hordei* still enhances acid production, but no white line develops. (D) In the absence of the white line precipitate, terrestric acid accumulates at the bacterial edge and *P. hordei* is able to invade and overgrow the *B. subtilis sfp* colony. (E) When acid production in *P. hordei* is highly reduced on LB-maltose agar, the fungus fails to precipitate *B. subtilis* LPs, resulting in increased inhibition of the fungal colony. Red triangles indicate plipastatin, orange dots indicate surfactin, green rings indicate terrestric acid, and brown pentagons indicate CA.

Cross-kingdom interactions between fungi and bacteria are crucial for community function and niche formation, yet the secondary metabolites mediating these interactions remain underexplored [6, 37]. Here, we investigated a secondary metabolite-mediated interaction between *B. subtilis*, a well-known plant beneficial bacterium, and the non-pathogenic rhizosphere-associated *P. hordei* [9, 38]. We show that this interaction leads to acid-driven precipitation of multiple *B. subtilis* lipopeptide families, forming insoluble aggregates that appear over time as a distinct white line on agar plates (Fig. 6A and B). While a similar “white line” phenomenon has been reported in *Pseudomonas*-*Pseudomonas* interactions and was hypothesized to result from the co-precipitation of cyclic LPs [39–42], our work provides the first evidence of this process occurring in a cross-kingdom interaction, where we also elucidate the underlying mechanism involving fungal organic acid secretion and the consequences for the growth and development of both interacting partners.

As previously reported for several interaction studies driven by SMs, both *P. hordei* and *B. subtilis* mobilize and modulate their metabolome upon perception of each other [33, 43–45]. Our findings show that *P. hordei* produces a suite of organic acids, particularly TA and its biosynthetic derivatives, which accumulate during growth and are further enhanced upon co-cultivation with *B. subtilis*. This acidification correlated with the appearance of LP precipitation, suggesting a fungal response stimulated by bacterial competition [46]. Consistent with this observation, when *P. hordei* was co-cultivated with *B. subtilis* under conditions that strongly reduce organic acid production (LB-maltose), the white line failed to develop, leading us to link acid production to the observed LP precipitation. This interpretation is reinforced by the fact that strong acidification is a well-established method for precipitation and purification of cyclic LPs in vitro, supporting the plausibility that fungal-derived organic acids drive LP aggregation in vitro [47, 48]. Mechanistically, acidic conditions alter the protonation states of amino acid residues in LPs, reducing their net charge. This loss of charge decreases water solubility, leading to precipitation, while also affecting self-assembly and aggregation, which further promotes clustering and precipitation [49, 50].

In turn, fungal acidification influenced the *B. subtilis* secondary metabolome. We observed a strong reduction in plipastatin production specifically under TA-mediated acidification, whereas other acids such as fulvic or barceloneic increased plipastatin levels. Given that plipastatins are potent antifungal LPs and central to *Bacillus* biocontrol activity, their suppression provides advantage for the fungus [51, 52]. Acidification-induced suppression of bioactive metabolites has been reported as a general advantage against antibiotic-producing microbes, whose compounds seem to be less active at acidic pH [53]. The fact that distinct acids had opposite effects suggests that *Bacillus* lipopeptide biosynthesis is not simply pH-sensitive but acid-specific, likely reflecting differential stress responses and regulatory pathways [54–56]. Previous studies have shown that *Bacillus* secondary metabolism is highly responsive and influenced by fungal competitors [57–59]. Our results extend this view by showing that fungi can modulate *Bacillus* antifungal capacity through the type and concentration of the organic acids they secrete.

Once we elucidated the chemical basis of the interaction, we found that the white line shapes the spatial dynamics and the outcomes (colony invasion, inhibition, or co-existence) of the *B. subtilis*–*P. hordei* interaction (Fig. 6B–E). MALDI-MSI showed that TA and its derivates diffuse toward the *B. subtilis* colony but halt at the precipitate, indicating that the white line forms a chemical boundary that restricts metabolite exchange and microbial colony expansion. When this barrier was weakened or absent, either through the lack of *Bacillus*-produced LPs or growing *P. hordei* in LB-maltose, the interaction shifted toward antagonism: *P. hordei* invades the bacterial colony or *Bacillus* inhibiting the fungus, respectively. We therefore proposed that LP precipitation functions as a physical–chemical barrier that structures the interaction and could also contribute to stabilizing fungal-bacterial coexistence. In the rhizosphere, chemically defined microenvironments may represent a niche partitioning mechanism, reducing direct competition and supporting the persistence of diverse communities [60].

Subsequently, we assessed how widespread the white line precipitation phenomenon is by broadening the set of interacting partners. On the fungal side, the white line formation was conserved among *Corymbifera* species through the production of TA, fulvic, or barceloneic acids, but also extended to non-*Corymbifera* producers of TA such as *P. crustosum* and *P. aurantiogriseum*. These findings suggest a phylogenetically restricted but chemically convergent trait, with TA at sufficient concentrations acting as a universal driver of *Bacillus* LP precipitation. This points to a broader distribution of the organic acid defensive strategy among *Penicillia* to counteract *Bacilli* and persist in the niche to exert potential beneficial effects on the plant.

On the bacterial side, *P. hordei* induced precipitation with *B. subtilis*, *B. velezensis*, and *B. tequilensis*, but not with any *Pseudomonas* strains tested. Even though we observed a strong medium acidification in all *Pseudomonas*-*P. hordei* interactions, no white line developed, and fungal growth remained unaffected. The differences between LP precipitation may arise from the distinct physicochemical properties of *Pseudomonas* cyclic LPs (e.g. viscosin and orfamide) compared to their *Bacillus* analogs. Although the *Pseudomonas* LPs also contain acidic side groups which protonate under low pH environments, they may tolerate acidic conditions better; *Pseudomonas* uses gluconic acid to mediate environmental acidification, which may buffer its LPs from precipitation at the pH level reached in the interaction with *P. hordei* [61, 62]. In addition, many *Pseudomonas* LPs are more hydrophilic, containing a higher proportion of non-charged hydrophilic amino acids, which helps maintain solubility under acidic conditions [63].

Altogether, our findings reveal a fungal defensive strategy by which *P. hordei* disarms *Bacillus* antagonism through acid-driven precipitation of LPs and the specific reduction of the plipastatin biosynthesis, a key bacterial antifungal metabolite. Whether this phenomenon occurs in the rhizosphere has yet to be determined, however, the mechanistic understanding determined here in the agar environment provides the basis for future investigations into the competitive role of these soil microbiota as well as the potential use of these biocontrol agents in highly acidic soils.

## Supplementary Material

SUPPLEMENTARY_INFORMATION_REVISED_ycaf172

Video_S1_ycaf172

Video_S2_ycaf172

## Data Availability

MSI data can be found at Metaspace at https://metaspace2020.org/project/vertot-2025. FBMN workflows can be found at the GNPS website under the following links: (1) *P. hordei*-*B. subtilis* co-culture on PDA (positive mode): https://gnps.ucsd.edu/ProteoSAFe/status.jsp?task=8e0f80d153d34f3b894f4eb36d3724ca; (2) *P. hordei*-*B. subtilis* co-culture on PDA (negative mode): https://gnps.ucsd.edu/ProteoSAFe/status.jsp?task=c32e6695bddf4b86aa7f3f6d8707a82c; (3) *Corymbifera*-*B. subtilis* interactions (positive mode): https://gnps.ucsd.edu/ProteoSAFe/status.jsp?task=67d33af1d1c0471ea1cb943a5be62099; (4) *Corymbifera*-*B. subtilis* interactions (negative mode): https://gnps.ucsd.edu/ProteoSAFe/status.jsp?task=7eab8907eb45410b9b4df511907ef765 (5) *P. hordei*-*B. subtilis* co-culture on LB maltose (positive mode):https://gnps.ucsd.edu/ProteoSAFe/status.jsp?task=02592e5e63344fe5a10d7463b559c777
