# Correction to: *Penicillium hordei* acidification precipitates *Bacillus subtilis* lipopeptides to evade inhibition

**DOI:** 10.1093/ismeco/ycag234

**Published:** 2026-09-17

This is a correction to: Manca Vertot, Morten D Schostag, Aaron J C Andersen, Jens C Frisvad, Carlos N Lozano-Andrade, Scott A Jarmusch, *Penicillium hordei* acidification precipitates *Bacillus subtilis* lipopeptides to evade inhibition, *ISME Communications*, Volume 5, Issue 1, January 2025, ycaf172, https://doi.org/10.1093/ismeco/ycaf172

The following change has been made to the originally published paper.

In section **Results**, text in the first subheading is corrected from: “*Puccinia hordei*” to read: “*Penicillium hordei*”.

